# The Artificial Intelligence in Digital Radiology: Part 1: The Challenges, Acceptance and Consensus

**DOI:** 10.3390/healthcare10030509

**Published:** 2022-03-10

**Authors:** Daniele Giansanti, Francesco Di Basilio

**Affiliations:** 1 Centro Tisp, Istituto Superiore di Sanità, 00161 Roma, Italy; 2 Facoltà di Medicina e Psicologia, Università Sapienza, 00185 Roma, Italy; fdibasilio@hotmail.com

**Keywords:** digital radiology, radiology, picture archive and communication system, artificial intelligence

## Abstract

Artificial intelligence is having important developments in the world of digital radiology also thanks to the boost given to the research sector by the COVID-19 pandemic. In the last two years, there was an important development of studies focused on both challenges and acceptance and consensus in the field of Artificial Intelligence. The challenges and acceptance and consensus are two strategic aspects in the development and integration of technologies in the health domain. The study conducted two narrative reviews by means of two parallel points of view to take stock both on the ongoing challenges and on initiatives conducted to face the acceptance and consensus in this area. The methodology of the review was based on: (I) search of PubMed and Scopus and (II) an eligibility assessment, using parameters with 5 levels of score. The results have: (a) highlighted and categorized the important challenges in place. (b) Illustrated the different types of studies conducted through original questionnaires. The study suggests for future research based on questionnaires a better calibration and inclusion of the challenges in place together with validation and administration paths at an international level.

## 1. Introduction

We are witnessing the introduction of Artificial Intelligence in many areas of medicine. Among these sectors, we find the sector of digital radiology and digital pathology. The potential is very large. Although with different speeds and degrees of use, Artificial Intelligence is proving useful in both sectors in many activities. The radiology and pathology *workflow* can certainly benefit from a routine use of Artificial Intelligence. The advantages that could derive from this seem important [1]. They range from lightening the workload to quality control of the instrumentation. The hospital routine, however, has some mandatory steps before the implementation. Among these passages, we certainly find those relating to *acceptance* and *consensus*. The *acceptance* and *consensus*, with regards to the Artificial Intelligence, are playing an increasing interest both in Digital Radiology and Digital Pathology and are among the topics of strong attention of the Special Issue “The Artificial Intelligence in Digital Pathology and Digital Radiology: Where Are We?” [2].

The recent experience of Artificial Intelligence in Digital Radiology, during the still active pandemic, seems promising. The successful applications of Artificial Intelligence in Chest Computerized Tomography/Radiography seem to bring the moment of the introduction in the clinical routine ever closer [3,4].

This will certainly happen through an adequate transfer of Evidence-Based Medicine to clinical practice and the *health domain, by means of agreement tools*. The Technology Assessment tools, such as the Health Technology Assessment and the comparative effectiveness research [5], will certainly be useful. Other methodologies, such as the Consensus Conference methodologies [6], will be able to be implemented. Consensus Conferences are currently used successfully in other emerging sectors using also Artificial Intelligence such as Robotics [7,8]. From future studies, useful guidelines for clinical dissemination will presumably emerge [9]. 

Artificial Intelligence is subject to continuous challenges in the integration process within the *health domain* [10]. To face a good process of integrating Artificial Intelligence with Digital Radiology, it is necessary first: (a) to always creating new challenges “separating Hope from Hype” and avoiding pitfalls [10]; (b) to ensure that processes of acceptance and consensus of the insiders accompany these challenges, who will have to do with these technologies in their *workflow*. Therefore, the challenges will have to be followed by integration processes in the *health domain*, by means of agreement tools based also on studies of *acceptance and consensus*. 

## 2. Purpose 

The purpose of the study was to focus on the Artificial Intelligence integration and:

(a) To examine the main challenges in the field in relation to integration in the health domain.
  This point is addressed to answer the key question “*What are the current challenges to be faced in integrating these technologies into the health domain?*”
(b) To deal with the topic of acceptance in the health domain and to address the relevant state of implementation of the used tools to assess this on the insiders.
  This point is addressed to answer the key question “*What tools are currently used to evaluate the acceptance of these technologies among insiders?*”
(c) As a side objective, to analyse how the challenges and acceptance are connected to each other and what are the possible ways to proceed to improve the integration of consensus among insiders. This point is addressed to answer the key question “*What suggestions emerge from the study to improve the tools used to assess acceptance among insiders, in light of what emerges in the previous points?*”

## 3. Methods 

According to the purpose, the study faced the three points of view.

Regarding the search of the studies, we considered that all points address the integration in the health domain. Therefore, we decided to orient ourselves first of all towards a database that contains international peer-reviewed studies in this area. 

We also considered the interdisciplinary aspects of the topic and we thought that the search had to be deepened also using a multidisciplinary database, always considering that the selection of the articles (see below) had to strongly consider the integration in the health domain. We have chosen PubMed (an archive focusing primarily on the life sciences and biomedical disciplines that contains Medline) as our main database. We have also expanded our search to include Scopus, a multidisciplinary database; however, strictly considering that we dealt with aspects of the health domain. 

The first two points faced literature searches, with targeted keys and through an eligibility process. The eligibility process was based on a scoring system (with different parameters and a score with 5 levels) applied by the two experts (plus one adjudicator in case of discordance), to include each reference. It is possible to assign a score to these parameters ranging from a minimum score of one (minimum) up to a maximum of five (maximun). Table 1 shows the scoring system.

The study was excluded, regardless of the score, if there were critical issues of conflict of interest (for example, it was conducted without guarantees of objectivity by the system manufacturer).

The study was included in the review if all parameters were scored ≥ 3.0.

The third point is a point of reflection, based on what emerges in the previous points, and therefore faced in the discussion.

The specific searches for the first two points are:

*(First point of view)* Search has been carried out in this area considering the key in Table 2.

*(Second point of view)* Search has been carried out in this area considering the keys in Table 3.

## 4. Results

### 4.1. The Challenges 

In line with the first objective of the study, we analysed the main challenges in the design and integration of Artificial Intelligence in digital radiology. The eligibility process led to the choice of 20 papers [11,12,13,14,15,16,17,18,19,20,21,22,23,24,25,26,27,28,29,30], among which there are mainly reviews (15 in number) (as it could be expected considering the broad topics covered) but also *5* recent scientific articles/focus articles on very specific aspects [15,26,27,29,30]. 

The search highlighted how:

- The picture that emerged was that of a scientific production touching all aspects relating to the challenges, from the challenges on algorithms [13] up to challenges on the ethical and legal issues [14].
- These challenges according to the following thematic analysis are arranged into six paragraphs with the synopsis of each paper.

#### 4.1.1. Challenges on Algorithms

A first study by Fazal et al. [11], on this topic, was relating to the evolution and perspectives of use of algorithms. The study showed that, after an initial difficulty, due to the limitation of the technology in 1960, the introduction of Artificial Intelligence-based computer-aided detection software in the 1980s marked the advent of widespread integration of Artificial Intelligence within radiology reporting. The authors pointed out among the macro areas of challenges in the algorithms: (a) to decrease the false-positive rates causing a limitation for computer-aided detection, although this has strongly improved. (b) The better understanding of the Artificial Intelligence reasoning: (c) The well definition of responsibility in case of the error caused by the algorithms. 

Another study by Maowad et al. [12] specifically focused on the challenges of machine learning and deep learning, subclasses of Artificial Intelligence that showed breakthrough performance in image analysis. The authors discussed the current applications of machine learning and deep learning in the field of diagnostic radiology. They highlighted that deep learning applications could be divided into medical imaging analysis and applications beyond analysis. The authors also highlighted how beyond image analysis, deep learning could be used for quality control, workflow organization, and reporting revolutionizing the activity of the insiders. 

The study by Barragan-Montero et al. [27] faced the challenges for a safe and efficient use of clinical Artificial Intelligence applications. They reported that this depended, in part, on informed practitioners and reviewed the pillars of Artificial Intelligence, together with state-of-the-art machine learning methods and their application to medical imaging. 

They metaphorically depicted that artificial intelligence, machine learning, and deep learning could be seen as *matryoshkas* nested in each other. Artificial intelligence gathered both symbolic (top–down) and connectionist (bottom–up) approaches. Machine learning was the dominant branch of connectionism, combining biological (neural networks) and statistical (data-driven learning theory) influences. Deep learning focused mainly on large-size neural networks, with functional specificities to process images. According to this, they reported a very fine mapping between different learning frameworks and strategies (together with some of the most popular algorithms or techniques used for each of them) and specific medical applications, tabling in details. This mapping was divided into three parts in the tabling: the basic learning frameworks (supervised, unsupervised and reinforcement learning); the hybrid learning frameworks blending supervised and unsupervised; and finally, common learning strategies solving consecutive learning problems or combining several models together. 

#### 4.1.2. Challenges Focused on the Professionalism of the Radiologist 

These innovations of Artificial Intelligence in Digital Radiology mainly revolve around the professional aspects of the radiologist. 

The study by Gampala el al. [16] highlighted the challenges around this professional at the end of the medical decision chain. Artificial Intelligence could simplify every activity, like ordering and scheduling, protocoling and acquisition, image interpretation, reporting, communication, and billing. Therefore, Artificial Intelligence could be useful both in the diagnosis (supporting the categorization activities with the image enhancement, feature assessment and recognition) and in the patient management and workflow. Therefore, the same way physicians were familiar with planning protocols or delineation guidelines, the clinical teams should start being familiar with guiding principles for data management and curation in the era of Artificial Intelligence. 

In line with this study, the study by Barragan-Montero et al. [27] highlighted how the Findability, Accessibility, Interoperability, and Reusability Data Principles [27] used in digital radiology must be rethought through challenges around the radiologist, including Artificial Intelligence.

The readjustment of these principles, according to the study by Banja et al. [26], encounters major challenges [26] regarding ethical and regulatory implications in the development and use of algorithms. All three studies [16,26,27] showed in a complementary way how, to support the radiologist, data science specialists should work on the development of increasingly performing and targeted algorithms, calibrated considering the specificity of the application, the decision-making protocols, and the physical process, which is different from time to time in the formation of images. For this reason, it is important that the radiologist talks with these scientific professionals, also involved in basic research, both to give new stimuli and to give feedback on use. 

#### 4.1.3. Challenges on the Tools, Datasets, and the Workflow

The challenges in Digital Radiology were also mentioned in the study by Ahmad [17], in the study by Hamed et al. [18], and in the study by Kottler [19]. Particular emphasis was dedicated to the challenges in the engineering and in the machine in terms of hardware, software, and impact on the workflow. These studies [17,18,19] also reported important new activities such as selecting Artificial Intelligence products and vendors; piloting vendors’ Artificial Intelligence algorithms; creating our own Artificial Intelligence algorithms; implementing, optimizing, and maintaining these algorithms. 

The study by Martin-Noguerol et al. [20] specifically focused on the challenges on the tools development. It reported the importance of the tools for the *use cases* and described them, including clinical registries, tools validation, and assistance for radiology reporting. In details, they reported a review of the tools required for successful implementation of the use cases.

The study by Cushnan et al. [28] emphasized the importance of the *use cases* and the experience of the National COVID-19 Chest Imaging Database, led by the British Society of Thoracic Imaging, Royal Surrey National Health Service Foundation Trust and Faculty in the collection of datasets on a national scale. This was a challenging experience to execute for several reasons, including issues with data privacy, the lack of data reporting standards, interoperable technologies, and distribution methods. The authors reported that this is a key issue to advance the safe adoption of artificial intelligence in the health domain.

#### 4.1.4. Challenges on the Teamwork 

A wide-range teamwork should work on the Artificial Intelligence development in digital radiology. The study by Martin-Noguerol et al. [20] reported the challenges in teamwork around these tools and, in particular, in the collaboration between engineers, systems developers and radiologists. The communication between radiologists and data scientists was considered crucial for successful collaborative work. There were emphasized the specific skills that are inherent to radiological and medical training, critical for identifying anatomical or clinical targets as well as for segmenting or labelling lesions. According to the authors, these skills would then have to be transferred, explained, and taught to the data science experts to facilitate their comprehension and integration algorithms. The study by Pesapane et al. [21] reported how the role of the stakeholders was also considered strategic in this team game. The authors reported that the stakeholders had the opinion that Artificial Intelligence could improve the practice of radiology and that they considered the replacement of radiologists unlikely. Furthermore, the study reported that stakeholders identified the need for education and training on Artificial Intelligence, as well as collaborative efforts to improve Artificial Intelligence implementation. 

#### 4.1.5. Challenges on the Education

The education and training were considered key factors for the integration of Artificial Intelligence in the health domain in different studies [15,22,23,29,30]. 

The study by Pesapane et al. [22] highlighted that the training needed to be continuous, specialized and based on a strong mobility on the territory, because it had to consider the continuous evolution of Artificial Intelligence.

The study by Pianiykh et al. [23] stressed the importance of continuous training, taking into account that network-based algorithms follow continuous learning processes [23]. The study by Fischetti et al. [30] showed how, with the integration of artificial intelligence within medicine, it was likely that the current medical trainee curricula could experience the impact it had to offer both for education and for medical practice. The study deepened the landscape of radiologic education within the current medical trainee curricula, and faced how artificial intelligence could potentially influence the current and future radiologic education model. From a specular point of view, the study by Reeder et al. [15] highlighted how Artificial Intelligence could also have a negative impact on the university choice of students. It underlined that Artificial Intelligence had a significantly negative impact on American medical students’ choice of radiology as a career, a phenomenon influenced by both individual concerns and exposure to Artificial Intelligence from the medical community. According to these authors, the challenge was also to avoid the impact of misinformation on Artificial Intelligence. In line with this, the study by Morrison et al. [29] reported the importance of education together with greater clarity of language. They concluded this by means of a thematic analysis that considered both the education and the clarity of language as favourable factors for eliminating the barriers to the adoption of Artificial Intelligence in the National Health Service. 

#### 4.1.6. Challenges on the Ethical and Regulatory Issues

Among the challenges, there were also those of the impact of ethical aspects [14,24,25] and of legal regulation [14,25]. In fact, with the growth in the use of Artificial Intelligence as a medical device, alone or interconnected with the network, the adaptation and compliance with the legislation was considered a fundamental challenge in terms of all aspects of use in the free market.

The study by Pesapane et al. [14] analysed the regulation in the context of medical device development, and the challenges to make Artificial Intelligence applications safe and useful in the future. The authors analysed the legal framework regulating medical devices and data protection in Europe and in the United States. The European Union was reforming these fields with new legislation (General Data Protection Regulation, Cybersecurity Directive, Medical Devices Regulation, In Vitro Diagnostic Medical Device Regulation). As regards the United States, the Food and Drug Administration predominantly controlled the regulatory scene. The study highlighted these fundamental aspects: -Artificial Intelligence applications were medical devices supporting detection/diagnosis, workflow, cost-effectiveness. -Regulations for safety, privacy protection, and ethical use of sensitive information were needed. -Europe and the United States had different approaches for approving and regulating new medical devices. -European laws considered cyberattacks, incidents (notification and minimisation), and service continuity. -Laws in the United Sates asked for opt-in data processing and use as well as for clear consumer consent. The study by Jaremko et al. [25] provided a framework for study of the legal and ethical issues on Artificial Intelligence in medical imaging, related to patient data (privacy, confidentiality, ownership, and sharing); algorithms (levels of autonomy, liability, and jurisprudence); practice (best practices and current legal framework); and finally, opportunities in Artificial Intelligence from the perspective of a universal healthcare system. The study by Akinci et al. [24] was entirely devoted to ethical aspects in radiology applications. The ethical issues were discussed under the light of core biomedical ethics principles and principles for Artificial Intelligence specific ethical challenges, while giving an overview of the statements that were proposed for the ethics of Artificial Intelligence applications in radiology.

### 4.2. The Tools Used to Assess the Acceptance

In line with the second objective of the study, we analysed the acceptance in the integration into the health domain. Many studies were excluded after a first rapid screening since the specific keys of research (e.g., acceptance) were not associated with content developed on this topic, and/or were associated with other contexts and/or cited in a single occurrence in a sentence. 

After this quick first screening, the eligibility process led to the choice of 15 papers [31,32,33,34,35,36,37,38,39,40,41,42,43,44].

The analysis was arranged into two paragraphs. The first one reports the general considerations that emerge in these studies. The second one reports a detailed analysis with the synopsis of each paper.

#### 4.2.1. General Considerations on the Tools for the Acceptance

The search highlighted:

(a) that the selected papers showed that the tools were essentially based on questionnaires [31,32,33,34,35,36,37,38,39,40,41,42,43,44];
(b) that the interest into this theme is recent, since publications of studies have started very recently, as the first ones are from 2019, and this reinforces the need for this study.

The selected papers [31,32,33,34,35,36,37,38,39,40,41,42,43,44] showed that the studies focused on some of the actors in this area: radiologists, radiographers, primary care providers, developers, students, and patients, that is, on both service providers and users, and on the subjects in training. 

In some cases, comparative studies were carried out on the different actors. 

Surveys were used to collect both interviews and structured data. In all identified cases, questionnaires based on *choice questions*, *Likert, graded questions* (in a psychometric scale)*, open questions* were used. 

The tools were nearly always based on original and not standardized questionnaires. Consolidated standardized tools currently used in radiology per technologies that have been consolidated for decades, such as the Picture Archive and Communication Systems [45] and the Technology Assessment Model, were not used, because they are unsuitable for unstable technologies undergoing evolution and development.

With very few exceptions, such as in [33], scholars preferred to use personal, original, and not validated/standardized questionnaires to investigate the topic. In fact, they focused on specific and new and never explored fields of the acceptance, from time to time different and not uniform (according to the felt need to produce medical knowledge), with the obvious impossibility of having specific tools ready and standardized to be reused, also considering the very recent interest in this field illustrated reported above. With very few exceptions, such as in [40,43,44], scientific societies were not involved.

#### 4.2.2. The Tools for the Acceptance in Details

Three studies [31,32,33] dealt with the questionnaires on *patients* (i.e., the customer of the service/final recipient). 

The first study by Fischetti et al. [31] focused on the integration of the Artificial Intelligence in the workflow. They collected opinions on several aspects of the use of Artificial Intelligence in the medical workflow from the patient entry up to the medical report. 

The second study by Zhang et al. [32] proposed interviews to extract considerations on the use of the Artificial Intelligence specifically in diagnostics. The considerations were positive. The study also suggested some concerns on cybersecurity. 

The third study by Ongega et al. [33] focused on the perceived perspectives on the Artificial Intelligence. The study showed: -the importance of the patients’ vision of Artificial Intelligence. -The impact of the social factors. -The usefulness of the questionnaires as sensors. 

The study reported by Hendrix et al. [34] focused on a first important actor (for the crucial role between the patient and the health domain): the *primary care provider*. The participants highlighted the importance of the sensitivity and other parameters in the clinical reports obtained with the Artificial Intelligence support. The use of Artificial Intelligence was considered adequate in a “triage” role to screen probable not positives without the need of the radiologist validation. 

The studies reported in [35,36,37,38,39,40] investigated the opinion of other insiders, including students during the training. 

The studies in [35,36,37] reported three investigations based on surveys submitted to *radiographers*. 

In details, the study by Abuzaid et al. [35] dealt with the acceptance in the workflow. The study showed: -a generalized enthusiasm for the integration of Artificial Intelligence in the training programs. -Interest for the potential of Artificial Intelligence. -Concerns on job security. -The importance of continuous education and training.

The study by Abuzaid et al. [36] dealt with the Magnetic Resonance Imaging applications. A focus group and a questionnaire were proposed to the participants. Participants thought that Artificial Intelligence could strongly improve the workflow. In addition, in this study, the importance of the continuous education and tuned training was remarked. 

The study by Giansanti et al. [37] collected opinions on the post-pandemic use of Artificial Intelligence and on the design of a structured questionnaire for the scientific societies. 

The study by Abuzaid et al. [38] investigated radiologists and radiographers’ opinions on the Artificial Intelligence integration into the radiology workflow. Results emphasized: -A low information on Artificial Intelligence with regards to the integration into the radiology workflow. -The importance of the design of appropriate training for both the radiographers and radiologists, with a careful consideration of the workflow. 

The study by Alelyani et al. [39] investigated the acceptance in terms of attitude on radiologists, radiographers, technologists, and students. The study underlined: -The awareness of the position of Artificial Intelligence in radiology. -A higher acculturation on the Artificial Intelligence of the radiologists. -The importance of introducing specific training in the courses at the medical schools.

The study by the European Society of Radiology [40] extended the investigation on the international scene. It reported the results of a questionnaire submitted to the members of the European Society of Radiology. Questions focused on the expectations in 5–10 years. Results highlighted: -A general favorable position on Artificial Intelligence. -Detailed information on the use of Artificial Intelligence. -Opinions on the responsibility. 

The study by Caparros Galan et al. [41] dealt with the opinion of the students. The participants were convinced that Artificial Intelligence could reform the radiology workflow. They did not think that this could have a dangerous impact on the work ability of radiologists. Also in this study, it was remarked the importance of the training programs on Artificial Intelligence.

The study by Di Basilio et al. [42], our companion paper (*Part 2*), proposed a questionnaire submitted to three different professionals, with different workflow backgrounds: the medical specialist, the medical physicist, and the specialist in data science. The study faced both the training and the various sectors of application of Artificial Intelligence in imaging and complementary activities. The study also highlighted the importance of the survey administration procedure, and two different methods were applied, with a different degree of interaction with the participants.

The studies in [43,44] reported two different survey administrations in this field, sponsored by two different scientific societies, and submitted on two different professionals. 

The first study by Diaz et al. [43] was an international survey on medical physicists, through the related scientific society.

The second study by Coppola et al. [44] was a nationwide survey conducted on radiologists by the Italian Society of Medical and Interventional Radiology.

The first study by Diaz et al. [43] dealt with the training aspects, the involvement in Artificial Intelligence projects or activities, with the opinion on the introduction of Artificial Intelligence, and on educational interests in this field. 

The second study by Coppola et al. [44] was mainly dedicated to the interaction with Artificial Intelligence (tasks by Artificial Intelligence, advantages, issues), the implications (ethical problems, risk of job loss, needs of policies), and to opinions. 

## 5. Discussion

A Sentiment analysis review conducted on Twitter showed an increasing attention on the integration of Artificial Intelligence in Digital Radiology [46]. Other review studies clearly showed that the pandemic represented an important engine for the development of this field [47,48,49] and an important lesson on how to continue for the future, as highlighted in the perspective reported in [37]. Other studies considered Artificial Intelligence in Digital Radiology in terms of impact to equity [50]. There was a [50] belief that Artificial Intelligence had the strength to either widen the health inequity divide or substantially reduce it. The authors [50] believed that, with a careful attention in the use: entirely realized, Artificial Intelligence integrated in the health domain could be a part of the broader strategy convergence on local, national, and global health equity.

We fully agree with these studies [46,47,48,49,50]. We believe that challenges and acceptance of the integration of technology must be interconnected and go hand in hand so that expectations are not disillusioned, and we can obtain Artificial Intelligence that offers us an increasingly effective and health-oriented useful approach. 

Our study in line with these considerations addressed two important points of view of the introduction of artificial intelligence in the health domain. 

The first point of view related to an analysis of the challenges in the development and integration of Artificial Intelligence in the health domain. 

The second point of view related to studies on the acceptance and consensus on the integration of Artificial Intelligence in the health domain mainly carried out through surveys. 

### 5.1. The First Point of View: The Challenges

The first point of view reviewed the challenges and grouped them into six main topics. The first topic is represented by the challenges in the design and employment of the algorithms [11,12,27], in their performance improvement [11], in their potential, not also limited to the imaging field [16], and their specific application based on the solution [27]. A second topic was the revolution of the workflow [16] of the radiologist; many activities could be possible in relation to imaging and many other activities in relation to the administration of other phases of the patient management process (i.e., patient scheduling). The third topic related to the new spreading IT tools that could be strategic in the health domain [17,18,19]. This determined (and hence the fourth topic) the need to intensify the collaboration between [19,20,21] the insiders. The fifth topic is the need of targeted training also including the mobility in the territory [22,30]. The latest topic is related to the consolidation of the Artificial Intelligence system as a medical device and the related regulatory framework, from which ethics and emerging risks could not be excluded [14,24,25].

### 5.2. Limits and Recommendation for Future Deepening on the Challenges 

Some limits and recommendation for future deepening (also because the argument was very broad and heterogeneous) emerged. 

If we dwell on the algorithms, it could be seen from the study in [27] how different specific solutions based on Artificial Intelligence algorithms (suitably categorized by the authors) were necessary for different applications. In subsequent studies, it will be necessary to deepen this theme based on the categorization proposed by this study. This categorizing certainly also had an impact on IT tools and training databases [17,18,19,28], which could be appropriately addressed with further specific studies. 

Particular attention in subsequent studies will need to be placed on the professional aspect of the radiologist and on other insiders. It will be necessary to address in detail all aspects of the change in the workflow in a specific way [16,26].

It will be essential to carry out studies on education, considering what has emerged [30], also bearing in mind the national programs. Distorting factors, cultural mediation, and the impact of training on the introduction of Artificial Intelligence will have to be investigated with also considering their relationships [15,29]. 

Finally, further studies will have to deepen the regulatory challenges (including also ethics) that now have been dealt with in a sectorial and patchy way [14,25]. 

### 5.3. The Second Point of View: The Tools for Investigating the Acceptance

The second point of view showed that, in the last two years, there was a notable development of studies addressing acceptance and consensus on the introduction of Artificial Intelligence in radiology using surveys [31,32,33,34,35,36,37,38,39,40,41,42,43,44]. The review showed that these studies considered different professionals (also with comparative studies between some professionals), such as radiologists, medical radiology technicians, primary care providers, students, and patients, that is, they focused on both the service providers and users, but also on subjects in training. The proposed surveys [31,32,34,35,36,37,38,39,40,41,42,43,44] were nearly always different and original from each other and led to very specific results. 

### 5.4. Limits and Recommendation for Future Deepening on the Tools for Acceptance

The study revealed various limitations and the need for future developments.

First, if we look at the studies, we see that the aspects related to the opportunities and challenges have been addressed but patchy, never together and not in detail in each questionnaire. Surveys should therefore be developed that address as much as possible the aspects highlighted in the first point of view. They focused on specific, new, and never-explored fields of the acceptance, from time to time different and not uniform (according to the felt need to produce medical knowledge). In consideration of this and on the very recent interest on this topic (the first publications are from 2019), scholars used nearly always not validated surveys. Validation paths for the surveys are recommended for the future, also to allow a common language to scholars. The support of scientific societies took place in limited cases and was not always based on international initiatives [40,43]. The federations of scientific societies should be more involved to provide greater support, as highlighted in [42], since Artificial Intelligence is a crosscutting issue affecting various areas. 

### 5.5. Limitation of the Study

The study was based on two narrative reviews dedicated to the two points of view. It analysed the scientific production in the English language as regards the publications. It did not analyse the publications in other languages (Spanish, French or Italian). The study used only two databases (PubMed and Scopus) and peer reviewed studies. Expansions of this type of survey could consider databases that include non-peer-reviewed conference articles and preprint sites. Preprint databases could give a further idea of how scholars are moving in this area by analysing articles undergoing peer review. The issue we have faced is wide-ranging and includes sub-themes that would require in-depth studies of specific studies (e.g., ethics, regulations, specificity of algorithms, just to name a few). We therefore preferred not to develop a systematic review that would have been difficult, complex and convoluted to implement, and we turned to other editorial categories admitted by the journal. Future developments could include systematic reviews targeted in the sub-themes identified.

## 6. Conclusions

There is great excitement around the introduction of Artificial Intelligence in the health domain and, in particular, in Digital Radiology. The pandemic seems to have given an important push towards the integration of Artificial Intelligence in Digital Radiology. It is also important that the challenges in this area be accompanied by actions in the direction of the integration of consensus conducted on insiders and citizens. The study conducted two narrative reviews, with two points of view, in parallel to take stock of both ongoing challenges and of initiatives conducted to face the acceptance and consensus in this area. The first point of view highlighted that the challenges were multifaceted and concerned various interconnected aspects. These aspects were: technological features, changes in workflows, improvement of teamwork (e.g., among data science experts and radiologists), the design of adequate training, cultural mediation actions to eliminate the factors affecting the integration, the development of adequate regulations concerning the use of Artificial Intelligence as a Medical Device in electronic health and mobile health and ethics. Survey tools based on questionnaire could be of support to monitor each of the aspects identified directly on the health domain. The second point of view showed how several studies were produced based essentially on original, non-standard, non-validated surveys, which addressed sectorial aspects on different professionals and on patients (almost always taken individually), often without the patronage of scientific societies and with local (not international) initiatives.

The study recognizes the usefulness of the questionnaire tools but suggests that they be better calibrated in order to better include all the ongoing challenges, the categories concerned, and the federations of scientific societies potentially involved. It is also desirable that future studies produce validated questionnaires that could be disseminated via international initiatives.

## Figures and Tables

**Table 1:** Parameters used for the qualification.

Parameter	Description	Score (1 = min; 5 = max)
1	Is the research design appropriate?	
2	Are the methods adequately described?	
3	Are the results clearly presented?	
4	Are the conclusions supported by results	
5	Added contribute to the field	
6	Topicality level of the study	
7	Focus on the health domain	

**Table 2:** Keys used in the search (also with plurals).

Key
*“artificial intelligence”[Title/Abstract] AND “radiology”[Title/Abstract] AND (“challenges”[Title/Abstract] OR “future research”[Title/Abstract] OR “integration”[Title/Abstract] OR “opportunity”[Title/Abstract] OR “future direction”[Title/Abstract])*

**Table 3:** Keys used in the search related to the second point of view (also with plurals).

Key
*“artificial intelligence”[Title/Abstract] AND (“radiology”[MeSH Terms] OR “radiology”[All Fields] OR “radiography”[MeSH Terms] OR “radiography”[All Fields] OR “radiology s”[All Fields]) AND (“accept”[All Fields] OR “acceptabilities”[All Fields] OR “acceptability”[All Fields] OR “acceptable”[All Fields] OR “acceptably”[All Fields] OR “acceptance”[All Fields] OR “acceptances”[All Fields] OR “acceptation”[All Fields] OR “accepted”[All Fields] OR “accepter”[All Fields] OR “accepters”[All Fields] OR “accepting”[All Fields] OR “accepts”[All Fields])*
*(“consensual”[All Fields] OR “consensually”[All Fields] OR “consensus”[MeSH Terms] OR “consensus”[All Fields]) AND “artificial intelligence”[Title/Abstract] AND “radiology”[Title/Abstract]*

## Data Availability

Not applicable.
